# Development of a structure-validated hypersexuality scale in Chinese university students

**DOI:** 10.1186/s12888-021-03362-4

**Published:** 2021-07-14

**Authors:** Yanli Jia, Xu Shao, Chanchan Shen, Wei Wang

**Affiliations:** 1 Department of Clinical Psychology and Psychiatry/ School of Public Health, Zhejiang University College of Medicine, Hangzhou, China; 2 Department of Psychology, Norwegian University of Science and Technology, NO-7491 Trondheim, Norway

**Keywords:** Exploratory structural equation modeling, Hypersexual experience, Principal component analysis, Structure-validated questionnaire

## Abstract

**Background:**

Hypersexuality is associated with many psychiatric disorders and creates enormous burden for the affected individual, family and society. There are numerous inventories measuring behavioral, emotional or stressful features of hypersexuality, we still need a structure-validated questionnaire to measure hypersexuality in a comprehensive way.

**Methods:**

Based on previous inventories and related clinical descriptions of hypersexuality, we designed a matrix of 72 items related to hypersexual experience, and we invited 282 heterosexual university students who experienced hypersexuality at least once life-long to answer the matrix.

**Results:**

Through exploratory factor analyses and exploratory structural equation modeling, we constructed a Hypersexuality Scale, with a satisfactory model structure of five factors (or scales, 4 items for each scale) of hypersexuality, and named them as the Negative Impact, Emotional Coping, Uncontrolled Behavior, Post-sex Regret, and Increased Interest. Most inter-correlations of these factors were significant but in low or medium levels in all participants. Male students scored significantly higher on Negative Impact and Increased Interest than females did.

**Conclusions:**

The five scales described in this study might help to understand hypersexuality, and the Hypersexuality Scale might be applied to the clinical conditions related to hypersexuality.

## Background

Hypersexuality, also named as sexual addiction, sexual compulsivity, compulsive sexual behavior or sexual impulsivity, is a phenomenon generally characterized by excessive and intense sexual drives, sexual fantasies, sexual cognitions, or sexual activities. It is related closely to the clinical distress and the impaired functioning in individuals’ life domains such as the social, study, occupational, physical, or emotional areas [1, 2]. For instance, the compulsive sexual behavior disorder is regarded as a subtype of impulse control disorders in the 11th version of the International Classifications of Diseases [3]. On the other hand, Kafka proposed diagnostic criteria for hypersexual disorder [4], but it was not included in the main diagnostic criteria systems such as the Diagnostic and Statistical Manual of Mental Disorders, 5th edition (DSM-5) criteria [5].

The hypersexual behaviors were linked with the compulsive masturbation (56%), pornography use (51%) and extramarital sex (21%) in one study [6]. It has been calculated that the prevalence of hypersexuality is approximately 2% among university students [7], 5% among American adults (a rough estimation) [8], 3.3% among adult outpatients [9], and 4.4% among adult psychiatric inpatients [10]. On the other hand, there was a male preponderance (more than 60%) in people with hypersexuality [7, 9, 11]. Men reported more masturbation, sexual partners, and problematic cybersex than women did [12], while hypersexual women were more involved in sexual risk behaviors, and worries about physical pain and harm [13]. Another study has shown that hypersexuality characterizes 10.3% of men and 7.0% women participants in USA [14].

The exact etiology of hypersexuality is not fully known up to present. Some clinical-based models such as the neurobiological etiology [15, 16], addiction model [17], psychodynamic theory [18], and so on, have been proposed, but none of them presents a clear explanation for hypersexuality. Hypersexuality is also a common syndrome in other mental disorders such as bipolar disorder [19, 20], and hypersexual patients have more psychiatric comorbidities, including anxiety, substance use, mood and personality disorders [21, 22]. A recent study also revealed that compared to healthy individuals, hypersexual men had higher rates of impulsivity, attachment difficulties, affective disorders, and maladaptive emotion regulation strategies [23]. Moreover, hypersexuality increased the risk of infectious diseases, such as the sexually transmitted diseases and the acquired immunodeficiency syndrome [24, 25].

Besides the individual-based Compulsive Sexual Behavioral Scale [26], there are many questionnaires targeting the measurement of hypersexuality from different angles [27]. A recent review also pointed out that they measure one or several aspects of hypersexuality in terms of its behavior, emotion or stress [28]. For instance, in these questionnaires, except for the Mood Disorder Questionnaire [29] and the Revised Mood and Sexuality Questionnaire [30], the number of items measuring hypersexuality responding to dysphoric mood and stress is either just one or none. The Internet Sex Screening Test [31] is a highly specialized one but it is merely applied to evaluate problematic sexual behavior on the Internet. The Revised Mood and Sexuality Questionnaire [30] is also a highly content-specific one, which aims to assess the sex-related emotions and mood states. On the other aspect, the Sexual Addiction Screening Test [32, 33] is limited in a specific scope in heterosexual men, and it has a lower internal consistency in women [27]. Overall, these questionnaires have some limitations in the content or in the applicable participants, that is, no single questionnaire offers a comprehensive measure of hypersexuality up to date.

Based on the previous literature, we believe that the measure of hypersexuality includes the following aspects, and an item-matrix measuring hypersexuality of these aspects is developed accordingly. Firstly, the negative impact of hypersexuality on individual’s life domain, for example the item that “My self-esteem has been negatively impacted by my sexual activities” is a simplification of the item “My self-respect, self-esteem, or self-confidence, has been negatively impacted by my sexual activities” which is similarly included in Hypersexual Behavior Consequences Scale [34]. Secondly, the sex-related communication, for example that “I have used sexual jokes or implications when communicating with others”, which is similar with the item that “I use sexual humor and innuendo with others while online” in Internet Sex Screening Test [31]. Thirdly, the abnormal sexual behavior, for example that “I have beat and kicked, or restrained my sexual partners”, which is similarly included in Compulsive Sexual Behavior Inventory [35]. Fourthly, the increased sexual interest and pornography consumption, for example that “I’m more interested in sex than usual”, which is similarly included in Mood Disorder Questionnaire [29]. Fifthly, the hypersexual behavior in response to stress and mood, for example that “I often use sex to cope with difficult feelings (e.g., worry, sadness, boredom, frustration, guilt, or shame)”, which is also similarly included in Hypersexual Disorder Screening Inventory [36]. Sixthly, cognition of hypersexuality, for example that “I feel that my sexual behaviors are not normal”, which is similar to the item “Do you ever feel your sexual behavior is not normal?” in Sexual Addiction Screening Test [32, 33]. Seventhly, the regret after the impulsive sexual behavior, for example that “When I feel anxious or stressed, I am likely to do something sexual that I regret later”, which is also similarly included in Revised Mood and Sexuality Questionnaire [30].

For the development of a measure of hypersexuality in our study, we would like to use the exploratory factor analysis and the exploratory structural equation modeling (ESEM) procedures in a nonclinical, university student sample. The ESEM, as a confirmatory tool that integrates the best features of exploratory and confirmatory factor analysis, has more potential advantages over the confirmatory factor analysis, with more remarkable flexibility, better goodness of fit, and more accurate factor correlation, and it also has a wide applicability to clinical measure-research [37]. In addition, the ESEM has been considered as more viable for plenty of items with a modest sample size [37]. In the current study, we have hypothesized that: 1) the hypersexuality includes several aspects: awareness of hypersexual activity, increased sexual interest, increased pornography consumption, increased emotional-coping with sex, abnormal sexual behavior, negative consequences of hypersexual activity, and regret after impulsive sexual activity, and 2) male participants (university students) express higher levels of hypersexuality than their female counterparts.

## Methods

### Participants

Altogether 1872 students were invited to the study, but only data from 282 participants were kept and analyzed (198 males, mean age: 21.07 years ±2.11 S.D., age range: 16–27 years; and 84 females, mean age: 21.38 ± 2.85, range: 18–37). These students had at least one hypersexual experience and the related distress feelings. There was no significant age difference between the two gender groups (the Student t = − 0.90, *p* = 0.37, 95% Confidence Interval: − 0.99 ~ 0.37). All participants were confirmed to have no prior history of psychiatric disorders, nor other organic brain or physical lesions severely impairing sexual functioning, and to be free from alcohol or drugs, according to the DSM-5 [5] by an experienced psychiatrist (WW). Participants abstained from pornographic materials or masturbation for at least 72 h prior to testing. The study protocol was approved by a local ethics committee, and all participants gave their written informed consents (guardians signed written informed consent for the young adolescents).

### Measures

Participants were asked to complete the matrix of 72 items regarding hypersexuality in a quiet room, using a 5-point Likert rating scale: 1 (very unlike me), 2 (moderately unlike me), 3 (somewhat unlike and like me), 4 (moderately like me), and 5 (very like me). As mentioned in Introduction, the matrix concerned aspects: 1) negative impact of hypersexuality on some domains, such as study, work or life, 18 items, 2) sex-related communication with others, 6 items, 3) abnormal sexual behavior, 14 items, 4) increased sexual interest and pornography consumption, 11 items, 5) emotional coping with sex, 6 items, 6) cognition of hypersexuality, 12 items, 7) regret after impulsive sexual activity, 5 items. These items were randomized before being presented to participants.

### Statistical analyses

Answers to the 72 items were subjected to the principal component factor analysis, using the Predictive Analytics Software Statistics, Release Version 22.0 (IBM SPSS Inc., Chicago, IL, USA). The factor loadings were rotated orthogonally via the varimax normalized methods. Items which were loaded less heavily (below 0.45) on a target factor, or cross-loaded heavily (over 0.30) on more than one factor were removed from subsequent analyses one-by-one. The procedure continued until no further item was needed to be removed. Then, model fits of the remaining data (i.e., components extracted as latent factors) were evaluated by ESEM using Mplus 7.11 [38]. In this procedure, we used maximum likelihood estimation and Geomin oblique rotation as default methods, and the following indices were used to identify the model fit: the χ^2^/ df, the comparative fit index, the Tucker-Lewis index, the Akaike information criterion, the Bayesian information criterion, the standardized root mean squared residual, and the root mean square error of approximation.

When factors and the related items were identified, the internal reliabilities as expressed in the coefficient H [39] for each factor were calculated. Moreover, the gender difference of each factor scores was submitted to two-way ANOVA (i.e., gender × factor score) plus the post-hoc Student t test. A *p* value less than 0.05 was considered as significant for group comparisons. The Pearson correlation test was applied to evaluate the relationships between these factors in all participants, and a p value less than 0.01 was considered as significant for a meaningful correlation.

## Results

Answers to the 72 items measuring the hypersexual experience were submitted to a principal component analysis first. Results of the pre-analysis check were acceptable (KMO = 0.86; the Bartlett test of sphericity = 9525.26; *p* = 0.00). Seventeen eigenvalues greater than 1.0 were identified, and the scree plot indicated a level-off from the sixth factor on. The first five were 14.59, 5.19, 3.99, 2.34 and 2.18 respectively, which altogether accounted for 39.28% of the total variance (the first four altogether accounted for 36.25%, and the first six 42.03%). Therefore, we extracted four-, five-, and six-factor models for further analyses.

Through the ESEM, several (i.e., four-, five-, and six-factor) models with different items were constructed, and their Mplus model fitting indices were computed (Table 1). Overall, five-factor model structure and its item distribution are the best among the models. We have developed a Hypersexual Scale (HYPS, Table 2) with 20 items (four items each factor), and subsequently named its five factors.

**Table 1:** Fit models of factors regarding hypersexual experience in 282 participants

Model	χ^**2**^/df	Comparative fit index	Tucker-Lewis index	Akaike information criterion	Bayesian information criterion	Standardized root mean squared residual	Root mean square error of approximation [90% Confidence Interval]
**Six-Factor (23 items)**	1.62	0.95	0.91	19,440.86	20,056.34	0.028	0.047 [0.035, 0.058]
**Five-Factor (20 items)**	1.63	0.96	0.92	16,658.21	17,131.66	0.028	0.047 [0.034, 0.060]
**Four-Factor (20 items)**	2.65	0.88	0.81	16,662.13	17,077.31	0.041	0.076 [0.066, 0.087]

**Table 2:** Factor loadings of the five-factor model with 20 items after the principal component factor analysis in 282 participants

Items	Factor 1	2	3	4	5
My self-esteem has been negatively impacted by my sexual activities.	**0.83**	0.07	0.06	0.12	−0.05
My self-confidence has been negatively impacted by my sexual activities.	**0.81**	0.10	−0.03	0.18	−0.06
My sexual activities have negatively affected my mental health (e.g. depression and stress).	**0.73**	0.03	0.05	0.24	0.16
Frequent and intense sexual fantasies, impulses and behaviors cause significant problems for me in social areas of my life.	**0.65**	0.14	0.27	0.09	0.11
When I feel sad or depressed, I masturbate on my own.	0.11	**0.80**	−0.03	−0.04	0.08
I often use sex to cope with difficult feelings (e.g., worry, sadness, boredom, frustration, guilt, or shame).	0.07	**0.75**	0.05	0.28	0.09
When I feel anxious or stressed, I masturbate on my own.	0.08	**0.75**	0.04	−0.10	0.11
I often use sex to deal with stress or problems in my life.	0.03	**0.71**	0.12	0.25	0.11
I have beat and kicked, or restrained my sexual partners.	0.05	−0.01	**0.74**	0.04	−0.03
I have a period of time when the number of my sexual partners increases significantly.	0.11	0.14	**0.71**	0.09	−0.08
I have forced someone against his or her will to have sex.	0.20	0.00	**0.69**	0.16	0.06
I have a period of time when the frequency of using sexual toys increases significantly.	−0.06	0.04	**0.68**	0.11	0.14
When I feel sad or depressed, I am likely to do something sexual that I regret later.	0.17	0.20	0.14	**0.78**	0.08
When I feel happy or cheerful, I am likely to do something sexual that I regret later.	0.12	0.02	0.10	**0.77**	0.02
When I feel anxious or stressed, I am likely to do something sexual that I regret later.	0.13	0.24	0.17	**0.73**	0.14
I do things sexually that are against my values and beliefs.	0.25	−0.10	0.05	**0.46**	0.02
I am more interested in sex than usual.	−0.01	0.03	0.00	0.09	**0.78**
I see more pornographic magazines and videos than usual.	0.17	0.17	0.10	0.03	**0.75**
I browse more sexual websites on the internet than usual.	−0.06	0.07	0.24	0.08	**0.72**
I am more interested in sex or have more thoughts about sex.	0.02	0.12	−0.24	0.02	**0.62**

Note: loadings higher than 0.50 were bolded for clarity

Factor 1 was called “Negative Impact”, which described adverse consequences in mental health and some life domains, such as in study, work, or social area. Factor 2 was called “Emotional Coping”, which reflected that individuals used sex to cope with personal emotion and stress, and some feelings when experiencing hypersexuality. Factor 3 was called “Uncontrolled Behavior”, which described that participants experienced sexual abuses and compulsive behaviors, and had sex with multiple sexual partners and more sexual toys. Factor 4 was called “Post-sex Regret”, which reflected that participants presented regret after engaging in the sexual activities resulted from positive or negative mood. Factor 5 was called “Increased Interest”, which described that participants experienced more sexual interest, sexual thoughts, and pornography utilizations.

Further, two-way ANOVA showed a significant difference in the five HYPS factor (scale) scores between the two groups (group effect, F [1, 280] = 5.52, *p* < 0.05, mean square effect = 139. 98). The post-hoc Student t test detected that the male students scored significantly higher than females did on HYPS Negative Impact (t = 2.52, *p* < 0.05) and Increasing Interest (t = 2.69, *p* < 0.01). The coefficient H values of HYPS five scales were acceptable, and their inter-correlations were significant but remained in a low or medium level (Table 3).

**Table 3:** Scale scores (means ± S.D.) of the Hypersexuality Scale in men (*n* = 198) and women (*n* = 84), and their internal reliabilities (in coefficient H) and inter-correlations in 282 participants

	Factor score				Coefficient H	Inter-correlation			
	Male	Female	95% Confidence Interval	Cohen’s d		F1	F2	F3	F4
**F1 (Negative Impact)**	8.49 ± 3.93	7.31 ± 3.44*	0.26 ~ 2.10	0.31	0.84				
**F2 (Emotional Coping)**	11.14 ± 4.04	10.11 ± 4.23	−0.02 ~ 2.09	0.25	0.84	0.23#			
**F3 (Uncontrolled Behavior)**	5.83 ± 2.84	5.57 ± 2.53	−0.44 ~ 0.97	0.09	0.80	0.22#	0.15		
**F4 (Post-sex Regret)**	9.27 ± 3.88	9.52 ± 4.09	−1.27 ~ 0.76	−0.06	0.79	0.43#	0.28#	0.31#	
**F5 (Increased Interest)**	12.18 ± 3.55	10.95 ± 3.38*	0.33 ~ 2.12	0.35	0.81	0.12	0.27#	0.09	0.18#

Note: * *p* < 0.05 vs. Female, # significant correlation at *p* < 0.01

## Discussion

Using exploratory factor analyses and ESEM on the 72 items regarding hypersexual experience, we have constructed a satisfactory model structure of five scales with 20 items (four items each), namely the Negative Impact, Emotional Coping, Uncontrolled Behavior, Post-sex regret, and Increased Interest. These scales had acceptable internal reliabilities and low or medium inter-correlations, which confirmed our first hypothesis. Besides, our results that the male students had higher HYPS Negative Impact and Increasing Interest supported our second hypothesis. Therefore, the scales might help measure hypersexuality in general people and clinical population, as its clinical application might be a further assistance to the clinically based assessments such as the Compulsive Sexual Behavior Disorder Scale [40].

The first HYPS scale, Negative Impact, reflecting the adverse consequences due to hypersexuality, includes the psychological distress and some interference in individuals’ lives, which was in line with previous findings. Hypersexual patients acknowledged that they experienced more concern about work, legal, social, and psychological consequences, and experienced significantly more psychoticism than healthy people did [6, 13]. With respect to interference in life areas, around half of people in an online survey who used online sexual materials more than 11 h a week, reported that their behaviors had interfered with their important life domains such as education, work, and social activities [41]. Another online survey also found that in men with hypersexual behaviors, more than three quarters of participants felt personal distress and suffered from impaired functioning in life domains due to hypersexual behaviors [42]. Other studies also indicated that the hypersexual behaviors jeopardized their romantic attachments and partner relationships [42, 43]. Moreover, previous findings that men had more severe hypersexual symptoms than women, and its severity level was usually related to the intrapersonal and interpersonal difficulties [12], were in accordance with our results that the male students scored higher on the Negative Impact scale.

The second scale, Emotional Coping, describes the sexual behaviors used to cope with emotion and stress experienced by participants, which was consistent with those descriptions by Kafka [4]. Negative emotions or psychological distress was identified as the center of hypersexuality networks [44]. Hypersexual men experienced more depression and sexual boredom [45], and patients who had more hypersexual behavior consequences were likely to report the elevated impulsivity, depression, anxiety, stress proneness and emotional dysregulation [34]. Moreover, the emotional dysregulation was positively correlated with the compulsive sexual behavior, which might lead to the onset of compulsive sexual behavior [46].

The third scale, Uncontrolled Behavior, includes a series of sexual behaviors that deviated from normal levels and social norms, which were similar to those reported previously: the hypersexual behaviors as masturbation, pornography, cybersex, telephone sex, strip clubs, and sexual behavior with consenting adults [11]. In women, the pornography use, masturbation frequency, and number of sex partners were significantly positive predictors of hypersexual behavior [47]. Moreover, in 97 patients with sexual addiction, there were 40*.*2% reporting pornography dependence, 30*.*9% compulsive masturbation, and 23.7% protracted promiscuity [48].

The fourth scale, Post-sex Regret, reflects a regret after engaging in some sexual behaviors, regardless of their life values or experienced emotional states, which were in accordance with a study showing that the sexual desire caused by anxiety and depression was positively associated with the likelihood of regret after sex [30]. Another study also found that among heterosexual couples, the increasing likelihood of regrettable sexual behaviors in negative mood states was a significant predictor of sexual infidelity [49]. Furthermore, people with hypersexual behaviors were more likely to experience shame [50–52].

The fifth scale, Increased Interest, describes the increase in sexual interest, sexual thoughts, and pornography utilizations an individual experienced, which was in line with a study showing that hypersexuality was positively associated with sexual excitation and arousal [53]. It has shown that the pornography consumption occupies a peripheral position in hypersexuality networks [44], and it is one of significantly positive predictors of hypersexual behavior in women [47]. Among heterosexual university students, men usually perceived more sexual intentions than what women intended to communicate, and that men were more straightforward in terms of expressing sexual interest than women did [54], which supports that the male students scored higher Increased Interest in the current study. Meanwhile, among both genders, problematic pornography consumption was positively associated with hypersexuality [55]. Notably, regarding pornography consumption, men with high sexual desire held positive attitudes, while those with hypersexuality held negative attitudes [45].

However, the current study has suffered from several limitations. Firstly, personality might influence the hypersexual reports, but we failed to record the personality traits in our participants. Secondly, our participants were heterosexual university students, whether the results could be generalized to people of other ages or to homosexual or bisexual individuals remains unclear. Thirdly, our measure is a self-report, which might suffer from the recall bias and cognitive bias since reporting hypersexuality is shameful [51, 56].

## Conclusions

Using exploratory factor analyses and more appropriate method ESEM in Chinese university students, we have developed a structure-validated hypersexuality scale with five factors, namely the Negative Impact, Emotional Coping, Uncontrolled Behavior, Post-sex regret, and Increased Interest, and have demonstrated that male students scored higher on Negative Impact and Increased Interest factors. Our results might help to understand the construct of hypersexuality, and the Hypersexuality Scale might be used as a screen test in the general population and be applied to the clinical settings related to hypersexuality.

## Data Availability

The datasets generated and analyzed during the current study are available from the corresponding author on reasonable request.

## Abbreviations

DSM-5: The Diagnostic and Statistical Manual of Mental Disorders, 5th edition
ESEM: The exploratory structural equation modeling
HYPS: The Hypersexual Scale
